# Phenolic Extract from *Aralia nudicaulis* L. Rhizomes Inhibits Cellular Oxidative Stresses

**DOI:** 10.3390/molecules26154458

**Published:** 2021-07-23

**Authors:** Quentin Lion, Andre Pichette, Mouadh Mihoub, Vakhtang Mshvildadze, Jean Legault

**Affiliations:** 1 Laboratoire D’Analyse et de Séparation des Essences Végétales (LASEVE), Département des Sciences Fondamentales, Université du Québec à Chicoutimi, 555 Boulevard de l’Université, Chicoutimi, QC G7H 2B1, Canada; Quentin.lion@uliege.be (Q.L.); Andre_Pichette@uqac.ca (A.P.); Mouadh.mihoub1@uqac.ca (M.M.); vakhtang_mshvildadze@uqac.ca (V.M.); 2 Centre de Recherche sur la Boréalie (CREB), Département des Sciences Fondamentales, Université du Québec à Chicoutimi, 555 Boulevard de l’Université, Chicoutimi, QC G7H 2B1, Canada

**Keywords:** *Aralia nudicaulis* L., phenolic acids, ROS, UV-B, IR-A, fibroblasts, enriched extract, quantitative analysis

## Abstract

UV-B and IR-A radiation are important inducers of biological changes in skin involving ROS generation. The overloading of antioxidant defense mechanisms by ROS production could lead to photoaging and photocarcinogenesis processes. Various traditional usages are reported for *Aralia nudicaulis* L. extracts, including treatment of dermatological disorders. Antioxidant and anti-inflammatory properties have already been reported for other *Aralia* species possibly due to the presence of phenolic compounds. However, the phenolic composition and the potential activity of *A. nudicaulis* rhizomes extract against oxidative stress and UV/IR damages have not been investigated. The main aims of this study were to prepare a fraction enriched in phenolic compounds (FEPC) from *A. nudicaulis* rhizomes, to identify its major phenolic compounds and to assess its potential for protective effects against oxidative stress induced by UV-B, IR-A or inflammation. A quantitative LC-MS study of FEPC shows that chlorogenic, caffeic and protocatechuic acids are the main phenolic compounds present, with concentrations of 15.6%, 15.3% and 4.8% of the total composition, respectively. With a validated analytical method, those compounds were quantified over different stages of the growing period. As for biological potential, first this extract demonstrates antioxidant and anti-inflammatory activities. Furthermore, ROS generation induced by IR-A and UV-B were strongly inhibited by *A. nudicaulis* extract, suggesting that *Aralia nudicaulis* L. rhizome extract could protect dermal cells against oxidative stress induced by UV-B and IR-A.

## 1. Introduction

Skin aging results from different endogenous as well as exogenous effects. Exogenous factors may include solar irradiation, air pollution, cigarette smoke, nutritional stress, lack of sleep or extreme temperatures as the skin aging exposome [1]. Despite all these factors, solar irradiation is still the main contributor to cutaneous aging and skin cancer. The solar spectrum is composed with ultraviolet radiation (UVR), infrared radiation (IR) and visible light.

UV-R is divided into UV-C (<290 nm), UV-B (290–315 nm) and UV-A (315–400 nm). UV-C is completely absorbed in the atmosphere, while UV-A and UV-B reach the Earth’s surface and promote vitamin D synthesis [2]. However, they are attributed many physiologically negative effects such as inflammation and skin aging processes. The involvement of UV-B in many skin disorders including skin cancer is well established. UV-B radiation could have direct effects on biomolecules; for example, the formation of cyclobutane pyrimidine dimers (CPDs), pyrimidine [3,4,5] or pyrimidone photodimers [3] or photoisomerization of *trans*- to *cis*-urocanic acid [5]. Moreover, UV-B causes indirect generation of reactive oxygen species (ROS) [4], although the exact cause of ROS induction by UV-B is not clearly known. Beak et al. showed that NADPH oxidase and COX may play a major role in the production of ROS [6]. Unexpectedly, Heck et al. found that catalase, an enzyme known for its antioxidant role, is heavily involved in the production of ROS when keratinocytes are exposed to UV-B [7]. Human skin is also exposed to IR from several natural as well as artificial sources. Recent epidemiological data and clinical observations indicate that IR radiation cannot be considered as totally innocuous to human skin [8], particularly in photoaging induced by ROS production [9]. In some studies, the molecular mechanisms involved in this process such as cellular signal transduction and gene expression have been characterized. IR radiation induces the synthesis of matrix metalloproteinase-1 (MMP-1) via the mitogen-activated protein kinase (MAPKs) signalling pathway initiated by an increased generation of mitochondrial reactive oxygen species (ROS) [8,9,10,11,12,13].

It could be established that the combined effect of UV and IR radiations plays an important role in ROS generation. This overproduction of ROS can overload antioxidant defense mechanisms, resulting in oxidative stress and oxidative photodamage of proteins and other macromolecules (lipids or nucleic acids) in the skin. These ROS are believed to be critical mediators of the photoaging and photocarcinogenesis processes [14,15,16]. ROS can modify proteins in tissue to form carbonyl derivatives, which accumulate in the papillary dermis of photodamaged skin [17]. Botanical antioxidants have been shown to be associated with reduced incidence of ROS-mediated photocarcinogenesis and photoaging [4,15]. Polyphenols, such as phenolic acid (benzoic and cinnamic acid) derivatives and flavonoids are widely distributed in the plant kingdom. Their common structures with phenol functions and a conjugated double bond system makes polyphenols active antioxidants. The compounds easily become oxidized and they turn into quinones. Intermediate forms could stabilize [18,19] free radicals from hydroxyl radicals, superoxide anion radicals or lipid radicals [20]. They can also react with different enzymes through chelation mechanisms.

The Araliaceae family is popularly known along with the *Panax* and *Eleutherococcus* genus for their interesting adaptogenic and immunostimulant activities [21,22]. Some species of the *Aralia* genus like *Aralia elata* and *Aralia cordata* have also been studied for various bioactive properties and are found in some pharmacopoeias. Anti-inflammatory and antioxidant effects were two of the main activities recorded for these species. In this case, the study of *Aralia nudicaulis* L. rhizomes is relevant and some analogies could be made with therapeutic species like *Aralia elata* or *Aralia cordata*. *A. nudicaulis*, also known as wild sarsaparilla, is an indigenous plant species in North America, which can be found abundantly from the west coast to the east coast, especially in boreal and mixed wood forests [23]. The traditional use of wild sarsaparilla as an herbal medicine by First Nations peoples has been recorded many times. According to the North American Ethnobotany Database, 79 matches were found by searching *A. nudicaulis* [24]. The reported properties of *A. nudicaulis* include wound healing, anti-infection, blood purification and liver protection activity [25]. Although *A. nudicaulis* is abundant in North America, only few studies have been conducted and have reported the anticancer [26] and antimycobacterial [27] activities of extracts of this species. Major constituents attributed to their activities in the literature, were terpenes [28] and polyacetylene [29] compounds, respectively.

In this work, a fraction enriched in phenolic compounds (FEPC) was obtained from the rhizomes of *A. nudicaulis*. Its antioxidant, anti-inflammatory and cytotoxic properties were investigated using in vitro cell culture models. On the basis of the obtained results, the inhibition of ROS production induced by UV-B and IR radiations on WS-1 fibroblast cells were also evaluated. After the identification of the *A. nudicaulis*-enriched extract composition, a correlation between the three major phenolic acids contents and its biological activity was established by a validated method. Finally, the three major compounds were also quantified over different growing period stages.

## 2. Results and Discussion

### 2.1. Extraction Yield and Purification Method

A methanolic extraction followed by a liquid-liquid partition with ethyl acetate were performed on dried *A. nudicaulis* rhizomes. Extraction yields for the methanolic and ethyl acetate extracts are respectively 7.3 and 1.1% of the dried biomass. The ethyl acetate extract was purified on a HP-20 Diaion^®^ column with a methanol:water gradient. The fraction obtained with the 50:50 eluent system constitutes the Fraction Enriched in Phenolic Compounds (FEPC), representing a 0.1% of the dried biomass yield. Then we confirmed the enrichment in phenolic compounds of this fraction with a Folin-Ciocalteu assay performed on different extracts taken from different stages of the purification. The crude methanol extract, the liquid/liquid EtOAc extract and the final FEPC were dosed and contain 5 ± 1%; 25 ± 3%; 61 ± 2% of phenolic compounds, respectively.

### 2.2. Identification and Optimization of LC-MS Conditions

**Figure 1:**
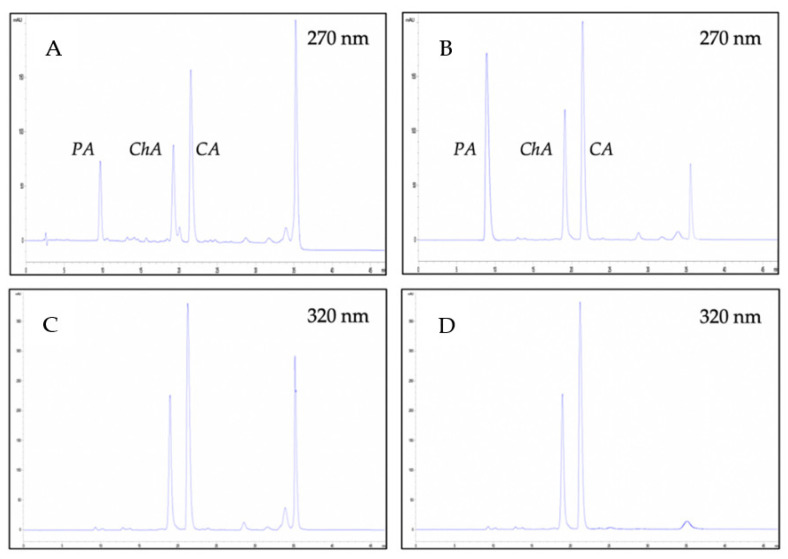
HPLC profiles of FEPC at 270 nm (**A**) and 320 nm (**C**); and HPLC profile of protocatechuic acid (PA), chlorogenic acid (ChA) and caffeic acid (CA) standards mix at 270 nm (**B**) and 320 nm (**D**).

A series of preliminary experiments were carried out in order to optimize the LC-MS conditions. Different mobile phases such as acetonitrile:water, methanol:water and acidified conditions were tested. The acidified mobile phase (0.1% formic acid) with methanol and gradient mode were necessary to achieve a satisfactory chromatographic separation in a reasonable period. Detection wavelengths were set according to the ultraviolet (UV) absorption maxima of the compounds (270 and 320 nm). In the FEPC extract, three main compounds were identified: protocatechuic acid, chlorogenic acid and caffeic acid. They were characterized and validated by comparing their accurate mass and retention times with those of standard compounds (Figure 1).

Many polyphenols are found in the *Aralia* genus, particularly flavonoids and flavonoid glycosides, but different phenolic acids have been found in *A. elata* and *A. cordata*, such as protocatechuic acid (PA), chlorogenic acid (ChA), caffeic acid (CA), vanillic acid, dicaffeoylquinic acid, neochlorogenic acid and cryptochlorogenic acid. PA and ChA and just ChA were quantified as major phenolic acids in *Aralia cordata* [30] and *A. elata* [31], respectively. With the same LC-MS method and standard comparisons, six other minor phenolic acids were found in FEPC: 1,3- and 3,5-dicaffeoylquinic acids, 3- and 5-feruloylquinic acids, *p*-coumaric acid and vanillic acid (Figure S1, Supplementary Materials).

### 2.3. Quantitative Method Validation

For the standardization of *A. nudicaulis* extracts, a quantitative method has to be optimized and validated. After examining different mobile phases and gradient tests, the acidified methanol:water binary gradient described in the Materials and Methods section was applied to proceed with the validation. The calibration results are summarized in Table 1, and a good correlation was found between the peak areas and the concentrations (R^2^ > 0.9995) for all standards in the range of concentration tested at their detection wavelengths. The limit of detection (LOD) is defined as the smallest peak detected while the limit of quantification (LOQ) is defined as the smallest peak quantified. In our work, detection and quantification limits were estimated by successively decreasing the concentration of the prepared standards to the smallest detectable peak. This concentration was multiplied by 3 and 10 to obtain the detection and quantification limits, respectively. As shown in Table 1, the LOD and LOQ were less than 0.014 and 0.043 mg/mL, respectively, which were low enough for the determination of the analytes in the phenolic compounds enriched extract from *Aralia nudicaulis*.

The repeatability and precision of the proposed method was evaluated using the relative standard deviation (RSD) of the peak areas of the three standards. These measurements of analysis repeatability are reported in Table 2 as inter-day and intra-day precision. In our work, the inter-day and intra-day tests were repeated 3 times within 1 day or between 3 successive days. RSD values for peak area were all ≤2.1% which is below the limit recommended by the International Conference on Harmonisation (ICH) guidelines [32].

### 2.4. Quantitative Analysis

The standardization method was firstly applied to quantify in *A. nudicaulis* extracts the three major phenolic acids: chlorogenic acid, caffeic acid and protocatechuic acid. As shown in Table 3, the contents and percentage of these compounds were greatly increased in the enriched fraction, representing 35.65% of the total composition. Protocatechuic acid content in all extracts was found in the range of 4.1 ± 0.6 in the methanolic extract to 48 ± 1 µg/mg in the FEPC. The protocatechuic acid content quantified in FEPC corresponded to 4.8% of the total composition. Chlorogenic acid and caffeic acid were found in the FEPC with a three times higher concentration than protocatechuic acid, at levels equivalent to 15.6% and 15.3% on the FEPC total composition, respectively. These results show an important concentration of these two phenolic acids, compared to 2.4% and 0.28% of chlorogenic acid and caffeic acid in the starting methanolic extract composition, respectively.

**Figure 2:**
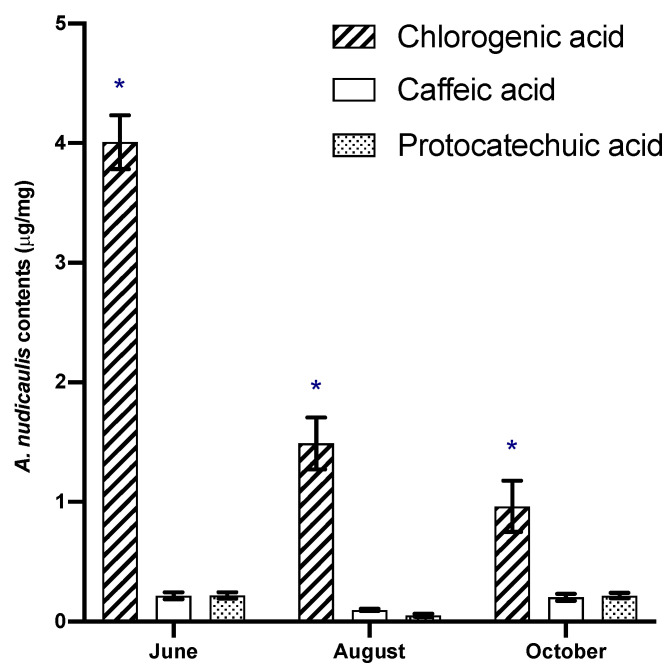
HPLC quantitative pattern of the three main phenolic acid (mean ± SD): chlorogenic acid (ChA), caffeic acid (CA) and protocatechuic acid (PA) during the growing season of *A. nudicaulis* L. Significative differences of each phenolic acids between the vegetation stages are indicated by (*) according to Duncan’s test (*p* < 0.05).

Chlorogenic acid, caffeic acid and protocatechuic acid were also quantified in crude extracts over the different vegetative stages of the plant. Concentrations were monitored at three different stages of the growing period: June (flowering), August (fruit-forming) and October (preparation for wintering). All data were expressed in Figure 2 as mean ± standard deviation, and ANOVA (post-hoc τ-test) was performed to compare the effects of phenolic acids and stages, using JMP^®^, version 14.1 (SAS Institute Inc., Cary, NC, USA). A *p*-value less than 0.05 was considered to be statistically significant (*).

As shown in Figure 2, *A. nudicaulis* crude extract analyses revealed chlorogenic acid as the major compound, especially at the beginning of the growing period. The ChA starts in June at 4.0 ± 0.2 µg/mg and decreases over the following months to reach 1.0 ± 0.2 µg/mg in October.

The concentrations of caffeic and protocatechuic acids demonstrate similarities in their variation pattern. At the beginning and the end of vegetation period the caffeic and protocatechuic acids contents were found to be in the range of 0.20 ± 0.03 to 0.22 ± 0.03 µg/mg. For these two phenolic acids, the lowest concentrations were detected at the fruit-forming phase, with 0.05 ± 0.02 and 0.10 ± 0.01 µg/mg, respectively, for caffeic and protocatechuic acids.

In Figure 2, a similar pattern can be observed for the variation of caffeic acid and protocatechuic acid, without any significant differences during growth. The significant differences (*) between the quantified compounds were the content and variation of chlorogenic acid (Figure 2). For this reason, the chlorogenic acid could be proposed as a chemical marker for *A. nudicaulis* crude extract standardization. In the *Aralia* genus, this is the first suggested standardization by quantification of phenolic compounds. Usually, terpenoid quantifications were performed to standardize *Aralia* extracts [33]. However, chlorogenic acid has been frequently used as a chemical marker for other extract standardizations [34].

### 2.5. Biological Activities Screening

A first screening of biological activities was performed with crude extracts, FEPC, phenolic standards and a mix of standards with a weight ratio based on the FEPC composition. This mixture of standards was used to mimic the FEPC to investigate the possible potentiation of biological activities due to a synergic effect of the extract.

To proceed to the following assays without any interactions, the cytotoxic activity of extracts and standards were evaluated against fibroblasts (WS-1), and the results are shown in Table 4. Results with FEPC were negative against WS-1, with an IC_50_ value of 87 ± 6 µg/mL for the resazurine test [35].

The antioxidant activity was assessed in vitro using the ORAC assay [36]. In Table 4, the results indicate that all tested species were strongly antioxidant, with ORAC values between 11 ± 1 and 18 ± 1 µmol Trolox/mg. In comparison, the ORAC value of quercetin, used as antioxidant standard, was 23 ± 3 µmol Trolox/mg. The antioxidant potential was also assessed in vitro using a cell-based assay [37]. Results for phenolic standards show IC_50_ values for the inhibition of *t*BH-induced oxidation of DCFH, between 0.12 ± 0.01 µg/mL and 0.13 ± 0.02 µg/mL. For FEPC and the mixture of standards the IC_50_ values are 0.31 ± 0.03 and 0.46 ± 0.03 µg/mL, respectively, compared with 0.27 ± 0.02 µg/mL for quercetin inhibition of DCFH oxidation. These results exhibit the strong antioxidant potential of the enriched *A. nudicaulis* extract. These results also support the hypothesis about the contribution of the three major phenolic acids in the antioxidant activity of the FEPC.

In a previous study [38], the anti-inflammatory activity of CA, the main compound of FEPC using LPS-stimulated RAW 264.7 macrophages was reported. Stimulation of RAW 264.7 macrophages by LPS induces iNOS and overproduction of nitric oxide (NO) [39]. NO released from cells can be detected and quantified photometrically as its stable product, nitrite, by a simple colorimetric reaction as described in the Materials and Methods section. l-NAME, a NO synthase inhibitor, prevents the formation of NO in LPS-stimulated RAW 264.7 macrophages and thus was used as positive control [40]. Two different concentrations of l-NAME were tested, 250 µM and 1 mM, with respectively 75 ± 9% and 89 ± 10% of inhibition of NO release. As expected, CA and FEPC show significant anti-inflammatory activities, with IC_50_ of 27 ± 14 and 35 ± 9 µg/mL, respectively. The low anti-inflammatory activities of other standards highlighted the major contribution of CA to the FEPC activity.

The decrease of the cytotoxicity against WS-1 between the methanolic extract and FEPC could be explained by the diminution of the terpenic contents [41] in the fraction enriched in phenolic compounds. The increase of phenolic contents, known for their antioxidant [42] and anti-inflammatory [43] potentials, could also cause the improvement of the different tested activities shown in Table 4.

In the *Aralia* genus, the antioxidant potential has been already recorded, particularly in *A. elata* [31], *A. continentalis* [44], *A. echinocaulis* [45] and *A. cordata* [30]. Antioxidant activities are often documented by a correlation with compositions rich in phenolic compounds. Moreover, anti-inflammatory activity has been also reported in *A. continentalis* [46], *A. elata* [47] and *A. echinocaulis*. In these *Aralia* species, the anti-inflammatory activities are mainly caused by diterpenoid and triterpenoid compounds.

### 2.6. Evaluation of FEPC Protection against UV-B and IR-A Radiations

**Figure 3:**
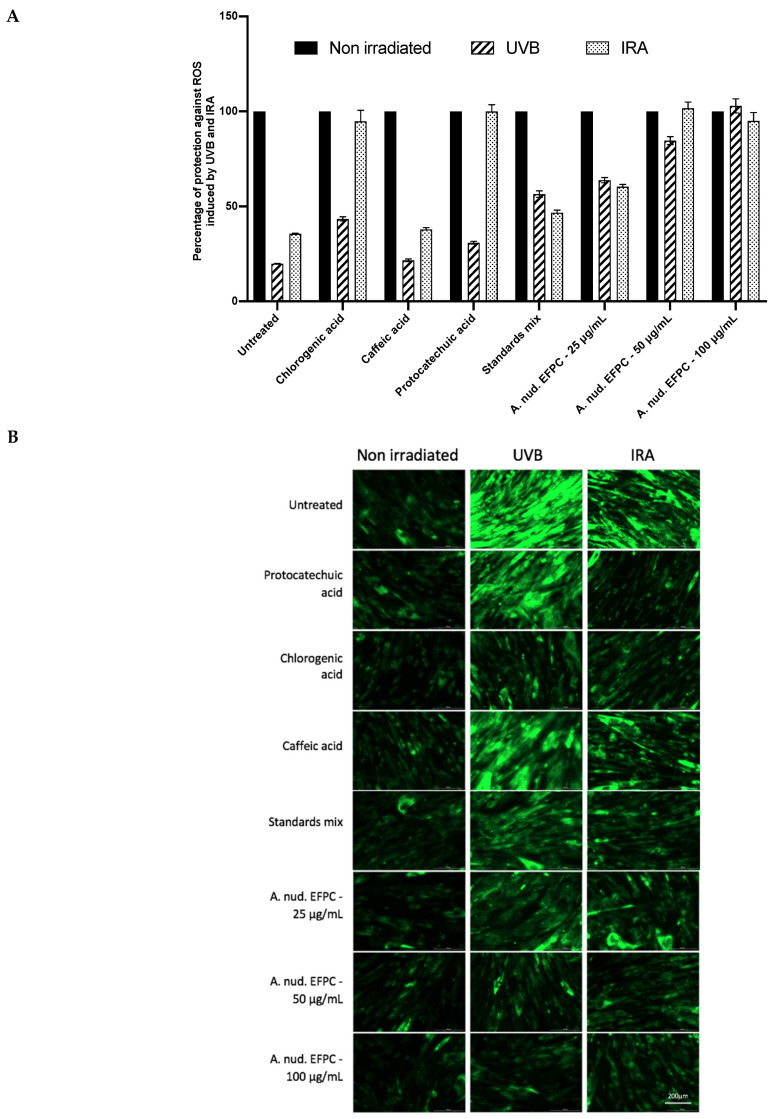
Evaluation of protective effect of FEPC and positive controls (**A**) following UV-B and IR-A irradiation (4700 mJ/cm^2^) on human skin fibroblast (WS-1). Inhibition of DHR-123 fluorescence (**B**) of irradiated-cells treated or not with FEPC, protocatechuic acid, chlorogenic acid and caffeic acid analysed by Image J software. This result is representative of three independent experiments.

The effect of FEPC in the protection of UV-B- and IR-A-irradiated human skin fibroblasts was evaluated as described above. Dihydrorhodamine 123 (DHR123) was used as a fluorescent probe to determine mitochondrial ROS induced by UV-B and IR-A radiations. In presence of ROS, DHR123 is oxidized to rhodamine 123 (RH123), and is progressively accumulated in the mitochondria. As shown in Figure 3A, the green fluorescence emitted by the oxidized RH123 makes it possible to evaluate ROS production that has occurred following cellular oxidations. Results show that WS-1 cells are weakly fluorescent if they are not irradiated. This basal fluorescence possibly corresponds to the ROS production during normal cellular metabolism. Treatment with FEPC or chlorogenic, protocatechuic and caffeic acids does not significantly change this fluorescence. After irradiation, the oxidation rate increases dramatically, especially after UV-B radiation.

Figure 3B shows that protocatechuic and chlorogenic acids are able to decrease the fluorescence by more than 88% in cells against ROS induced by IR-A, while their protection rates do not exceed 43% against UV-B. The protection rate of the caffeic acid does not exceed 37% for both types of radiations. The FEPC fraction shows a protection rate that exceeds 80% and 70% for UV-B- and IR-A-induced ROS at a concentration of 50 µg/mL.

In *A. elata* ethanolic extract, suppressive effects on UV-B-induced oxidative stress have been recorded, mainly caused by flavonoid glycosides [48]. Furthermore, inhibition effects on UV-B-induced oxidative or inflammatory mechanisms have been measured with chlorogenic [49] and caffeic [50] acids. Not many studies have evaluated similar effects for IR-induced stress. Nevertheless, as explained in the Introduction, polyphenols are known for reducing the incidence of ROS-mediated diseases.

## 3. Materials and Methods

### 3.1. Crude Extract and Enriched Fraction in Phenolic Compounds

*Aralia nudicaulis* L. *(A. nudicaulis*) rhizomes were collected from the boreal forest at Saguenay in northern Québec (Canada) recording the date of collection and the GPS coordinates (48.245498, −71.253504). The botanist P. Nadeau from UQAC made the botanical identification of *A. nudicaulis* and a voucher specimen (0297432) was deposited at the Louis-Marie Herbarium, Laval University (QC, Canada). The dried (air-dried protected from direct light) rhizomes were reduced to powder using a laboratory grinder. The crushed rhizomes of *A. nudicaulis* were stored at −20 °C. Plant material was extracted firstly with hexane to remove some lipophilic compounds, followed with three extractions with MeOH under reflux heating for 1.5 h. The obtained dry methanolic extract was dissolved in water and partitioned with EtOAc. The organic phase enriched in phenolic compounds was subjected to liquid phase chromatography on a HP-20 Diaion^®^ column and eluted with MeOH:H_2_O under gradient conditions (0% MeOH to 100% MeOH). All the chemical and biological tests were performed on the fraction obtained using 50% MeOH, that showed the richest phenolic composition (FEPC). For sample solutions, 40.0 mg of the phenolic compounds enriched fraction from *Aralia nudicaulis* roots was placed in a 10 mL volumetric flask and solubilised in HPLC grade MeOH. Three solutions were subsequently prepared by dilution (1.0; 2.0 and 4.0 mg/mL). Then 2 mL of each solution were filtered through a 0.45 μm membrane filter (Millipore, Oakville, ON, Canada, ref HVPL04700) before HPLC analysis.

### 3.2. Standardization Extract and Standards Preparation

*A. nudicaulis* rhizomes were collected at the same site three times during the growing season (in June, August and October). Immediately, the rhizomes were frozen in liquid nitrogen, lyophilized and powdered. Rhizome powder (0.5 g) was sonicated in 10 mL 70% aqueous methanol for 15 min in a FS220D 40 kHz ultrasonic cleaning bath (Fisher Scientific, Ottawa, ON, Canada), according to the method of Tian et al. [51]. After a centrifugation at 4000 rpm for 10 min, the supernatant was removed and the extraction was repeated twice more using the same method. Supernatants of the same sample were combined and evaporated. The obtained crude extract was resuspended in a 2 mL volumetric flask with MeOH and filtered through a 0.45 μm membrane filter (Millipore, Oakville, ON, Canada, ref HVPL04700) before HPLC analysis.

Stock solutions of 1 mg/mL of phenolic acid standards were prepared by dissolving the appropriate amount of each in a 10 mL volumetric flask with MeOH. All solutions were kept at −15 °C. The working standard solutions were subsequently prepared by dilution (in the concentration range of 0.01 to 0.5 μg/mL (*n* = 6). All prepared standard solutions were filtered through a 0.45 μm membrane filter (Millipore, Oakville, ON, Canada, ref HVPL04700) before HPLC analysis.

### 3.3. Chromatographic Instrumentation and Conditions

An LC series 1100 LC-MS system from Agilent Technologies (Mississauga, ON, Canada) equipped with a quaternary gradient pump, a vacuum degasser, a thermostated column compartment, an auto-sampler and a photodiode array detection systeusing by the Agilent ChemStation software. Reverse-phase chromatography was performed with an analytical C18 column (Kinetex, Torrance, CA, USA, 250 × 4.6 mm; 5 µm particle size), thermostated at 25 °C for the separation. The MS setting was in negative mode with electrospray (ES) and APCI ionization. All solvents used during this study, were HPLC grade solvents.

The optimized method used a binary-gradient mobile phase with water containing 0.1% formic acid as mobile phase A and MeOH as mobile phase B. The separation was performed with a gradient elution with the following proportions (*v*/*v*) of solvent B: 0–10 min, 12–16%; 10–11 min, 16–20%; 11–31 min, 20–24%; 31–32 min, 24–100%; and 32–37 min, 100%, at the flow rate of 1.0 mL/min, and the injection volume of 5 μL. Detection and quantification was carried out at 270 nm for protocatechuic acid; and at 320 nm for chlorogenic and caffeic acids.

### 3.4. HPLC Method Validation

The method was validated with standards in terms of linearity, intra-day and inter-day precision and accuracy. The linearity of the HPLC method was determined with six different concentrations of standards prepared and analyzed in triplicate for each concentration in the 0.01 to 0.5 mg/mL range. Calibration curves were constructed by plotting peak areas (U.A) against concentrations (mg/mL). The linearity was assessed by calculating the slope, y-intercept and coefficient of correlation (R^2^) using least squares regression. The calculations for the limits of detection (LOD) were based on the standard deviation of y-intercepts of the regression lines (σ) and the slope (S), using the following equation LOD = 3.3 σ/S. Limits of quantitation (LOQ) were calculated by the equation LOQ = 10 σ/S.

The precision of the method was evaluated with respect to both intra-day and inter-day precision. Three replicates of each level of phenolic acid standards were assayed in one run for the intraday experiment. Three replicates of each level of phenolic acid standards were assayed within three different days for the inter-day experiment. The intra-day and inter-day precision and accuracy of the assay were determined by the relative standard deviation (RSD). The accuracy of the method was evaluated using the recovery test. At each level, samples were analyzed in triplicates according to the previously described chromatographic conditions.

### 3.5. Measurement of Different Biological Activities

#### 3.5.1. ORAC_FL_ Assay

The procedure was modified from the method described by Ou et al. [36]. Briefly, the ORAC assay was carried out on a Fluoroskan Ascent FL™ plate reader (Thermo Labsystems, Waltham, MA, USA). Trolox was used as a control standard. The experiment was conducted at 37.5 °C and pH 7.4, with a blank sample in parallel. The fluorimeter was programmed to record the fluorescence of fluorescein every 1 min after addition of 2,2′-azobis(2-amidinopropane) dihydrochloride (AAPH). The final results were calculated by comparing the net areas under the fluorescein decay curves between the blank and the samples. ORAC values were expressed in micromoles of Trolox equivalents (TE) per milligram (μmol TE/mg).

#### 3.5.2. Antioxidant Cell Assay Using 2′,7′-Dichlorofluorescin-Diacetate (DCFH-DA)

Antioxidant activity was evaluated using the DCFH-DA assay as described by Girard-Lalancette et al. [37], with some modifications. Briefly, Human Skin WS-1 fibroblasts (ATCC CRL-1502, ATCC, Manassass, VA, USA) were plated in 96 microwell plates at 10,000 cells per well and incubated for 24 h at 37 °C and 5% CO_2_. The cells were washed with 150 μL Hank’s balanced salt solution (HBSS) at pH 7.4 and incubated for 30 min with 100 μL HBSS (pH 7.4) containing 5 μM DCFH-DA (Sigma–Aldrich, Oakville, ON, Canada). The cells were then washed again with 150 μL HBSS. To assess antioxidant activity, the cells were incubated either with a growing concentration of enriched fraction from *A. nudicaulis*, trolox or quercetin, in the absence or presence of 200 μM *tert*-butylhydroperoxide (*t*BH). Fluorescence was measured after 1 h and 4 h on the Fluoroskan Ascent FL™ automated plate reader using an excitation wavelength of 485 nm and an emission wavelength of 530 nm.

#### 3.5.3. Measurement of Anti-Inflammatory Activity by Nitrite Quantification

Exponentially growing RAW 264.7 (murine macrophages) were plated in 96-well microplates (BD Falcon, Franklin Lakes, NJ, USA) at a density of 7.5 × 10^4^ cells per well in 100 μL of culture medium (DMEM) and were allowed to adhere overnight. Cells were then treated with or without positive control *N*(w)-nitro-l-arginine methyl ester (l-NAME, ≥98%, Sigma–Aldrich), or increasing concentrations of extracts dissolved in DMSO, while the final concentration of solvent in the culture medium was maintained at 0.5% (*v*/*v*) to avoid solvent toxicity. Cells were then stimulated with 100 μg/mL lipopolysaccharide (LPS) and incubated at 37 °C, 5% CO_2_ for 24 h. After 24 h, cell-free supernatants were collected and immediately determined using the Griess reaction [52] with minor modifications. Briefly, 100 μL aliquots of cell supernatants were incubated with 50 μL of 1% sulphanilamide and 50 μL of 0.1% *N*-1-naphtylethylenediamine dihydrochloride in 2.5% H_3_PO_4_ at room temperature for 20 min. Absorbance at 540 nm was then measured using the automated Varioskan Ascent plate reader (Thermo Electron, Waltham, MA, USA) and the presence of nitrite was quantified by comparison with a NaNO_2_ standard curve.

#### 3.5.4. Evaluation of Cytotoxicity against WS-1 Cell Lines

Exponentially growing WS-1 cells were plated in 96-well microplates (Costar, Corning Inc., Tewksbury, MA, USA) at a density of 5 × 10^3^ cells per well in 100 µL of culture medium (DMEM supplemented with 10% foetal bovine serum, vitamins 1×, penicillin and streptomycin) and were allowed to adhere for 16 h before treatment. A concentration gradient of each compound was prepared in biotech grade DMSO (Sigma–Aldrich, Oakville, ON, Canada) and then diluted in DMEM before it was added to microplates (100 µL per well). Cells were then incubated for 48 h. The final concentration of DMSO in the culture medium was maintained at 0.5% (*v*/*v*) to avoid solvent toxicity. As described by O’Brien et al. [35], cytotoxicity was assessed using resazurin on an automated Fluoroskan Ascent FL^TM^ plate reader using excitation and emission wavelengths of 530 and 590 nm, respectively. Fluorescence was proportional to the cellular metabolic activity in each well. Survival percentage was defined as the fluorescence in experimental wells compared to that in control wells after subtraction of blank values.

#### 3.5.5. UV-B and IR-A Stress Protection Assay

WS-1 human skin fibroblast cells were plated in transparent flat bottom 96-well microplates (Greiner, µClear^®^, Kremsmünster, Austria) at 14,000 cells per well and incubated for 24 h at 37 °C and 5% CO_2_. Cells were treated (or untreated) during 20 min with 50 µL of FEPC sample at 100, 50 and 25 μg/mL or positive controls, namely, protocatechuic acid, chlorogenic acid and caffeic acid at 5, 15 and 15 μg/mL respectively. Cells were then exposed 4700 mJ/cm^2^ UV-B and incubated for 15 min. 50 μL of DHR-123 (1 μg/mL) was added to estimate ROS formed following radiations. After 5 min of incubation, cells were washed with PBS and observed on a cell imaging multimode reader using excitation and emission wavelength of 507 and 529 nm respectively. The same approach was followed for irradiation with IR-A, which was carried out under an infrared water-filtered lamp. The energy received by the cells has been estimated at 5300 mJ/cm^2^. Green fluorescence was quantified with ImageJ software. Each experiment was carried out in triplicate and the results are representative of at least three different experiments. Statistical analysis was carried out by the Kruskal-Wallis One Way Test followed by post-hoc Student-Newman-Keuls’ test using the SigmaStat 3.5 software (Systat, Palo Alto, CA, USA). *p* ≤ 0.05 was considered as significantly different.

## 4. Conclusions

The chemical composition of a phenolic extract from *Aralia nudicaulis* L. rhizomes was identified by LC-MS, and chlorogenic, caffeic and protocatechuic acids were the main compounds identified the extract. This fraction enriched in phenolic compounds (FEPC) from *Aralia nudicaulis* L. efficiently protects human skin fibroblasts against ROS induced by UV-B, IR-A and inflammation suggesting that this extract could be used as photoprotective agents. Moreover, the presence of phenolic acids in *Aralia nudicaulis* L. rhizomes could also partly explain its traditional uses for skin disorders. However, future studies will be needed to better understand the antioxidant mechanisms involved and also to confirm the protective effect on ex-vivo skin models.

## Figures and Tables

**Table 1:** Calibration curves, linearity, limits of detection (LOD) and limits of quantification (LOQ).

Analytes	Regression Equation	Linear Range (mg/mL)	Correlation Coefficient (R^2^)	LOD (mg/mL)	LOQ (mg/mL)
Protocatechuic acid	y = (13,871 ± 99.732)x − (22.992 ± 10.331)	0.030–0.500	0.999	0.1	0.029
Chlorogenic acid	y = (14,364 ± 103.94)x − (20.592 ± 11.008)	0.050–0.500	0.999	0.014	0.043
Caffeic acid	y = (26,397 ± 463.86)x − (7.5724 ± 23.462)	0.030–0.500	0.999	0.009	0.027

**Table 2:** Intra-day and inter-day precision and accuracy results.

Compound	Intra-Day (RSD, *n* = 3) %			Inter-Day (RSD, *n* = 3) %		
	0.01 mg/mL	0.05 mg/mL	0.5 mg/mL	0.01 mg/mL	0.05 mg/mL	0.5 mg/mL
Protocatechuic acid	0.40	0.41	0.24	0.70	0.62	0.20
Chlorogenic acid	0.81	1.4	1.3	0.60	0.81	0.81
Caffeic acid	0.64	1.1	2.1	0.43	1.3	0.54

**Table 3:** Quantification of major phenolic acids in the extracts from *A. nudicaulis*.

	Methanolic Extract (μg/mg; *w*/*w*%)	Ethyl Acetate Extract (μg/mg; *w*/*w*%)	FEPC (μg/mg; *w*/*w*%)
Protocatechuic acid	4.1 ± 0.6 (0.4%)	23.0 ± 0.7 (2.3%)	48 ± 1 (4.8%)
Chlorogenic acid	24.0 ± 0.3 (2.4%)	25 ± 2 (2.5%)	156 ± 1 (15.6%)
Caffeic acid	2.80 ± 0.09 (0.28%)	18.0 ± 0.2 (1.8%)	153 ± 2 (15.3%)

**Table 4:** Cytotoxic, antioxidant (WS-1 cells) and anti-inflammatory (RAW 264.7 macrophages) activities of FEPC and phenolic standards.

	Cytotoxicity IC_50_ (μg/mL)	ORAC (μmol Trolox/mg)	Antioxidant IC_50_ (μg/mL)	Anti-Inflammatory IC_50_ (μg/mL)
Methanolic extract	20 ± 1	0.09 ± 0.01	145 ± 48	>160
Ethyl acetate extract	30 ± 5	5.7 ± 0.7	0.38 ± 0.04	>160
FEPC	87 ± 6	12 ± 1	0.31 ± 0.03	35 ± 9
Protocatechuic acid	Nd	12 ± 1	0.13 ± 0.02	193 ± 58
Chlorogenic acid	Nd	11 ± 1	0.12 ± 0.01	89 ± 1
Caffeic acid	Nd	18 ± 1	0.13 ± 0.01	27 ± 14
Mix of standards	Nd	12 ± 2	0.46 ± 0.03	>160
Quercetin	Nd	23 ± 3	0.27 ± 0.02	Nd
l-NAME 250 μM	Nd	Nd	Nd	75 ± 9%
l-NAME 1 mM	Nd	Nd	Nd	89 ± 10%

IC_50_: Concentration inhibiting fifty percent of cells growth (cytotoxicity); DCFH oxidation (antioxidant activity) or NO overproduction (anti-inflammatory activity). Nd: Not determined.

## Data Availability

Data sharing is not applicable.

## Supplementary Materials

The following are available online, Figure S1: HPLC profiles of FEPC with composition identification, Table S1: HPLC quantification of FEPC compounds.

## Sample Availability

Samples of extracts are available from the authors.
