# Supplementary material for: Phenolic Extract from Aralia nudicaulis L. Rhizomes Inhibits Cellular Oxidative Stresses
Source: Molecules. 2021 Jul 23;26(15):4458. doi: 10.3390/molecules26154458 (PMC8347711; doi:10.3390/molecules26154458)
Supplement: Supplementary file 1 [file molecules-26-04458-s001.zip › molecules-1280960-supplementary.pdf]

molecules

MDPI

Supplementary Material

Phenolic Extract from *Aralia nudicaulis* L. Rhizomes Inhibits Cellular Oxidative Stresses

Quentin Lion [1], Andre Pichette [1,2], Mouadh Mihoub [1], Vakhtang Mshvildadze [1,2] and Jean Legault [1,2,*]

[1] Laboratoire d'analyse et de séparation des essences végétales (LASEVE), Département des Sciences Fondamentales, Université du Québec à Chicoutimi, 555 Boulevard de l'Université, Chicoutimi, QC G7H 2B1, Canada; Quentin.lion@uliege.be (Q.L.); Andre_Pichette@uqac.ca (A.P.); Mouadh.mihoub1@uqac.ca (M.M.); vakhtang_mshvildadze@uqac.ca (V.M.)

[2] Centre de Recherche sur la Boréalie (CREB), Département des Sciences Fondamentales, Université du Québec à Chicoutimi, 555 Boulevard de l'Université, Chicoutimi, QC G7H 2B1, Canada

* Correspondence: Jean.Legault@uqac.ca; Tel.: +14-18-545-5011 (ext. 2334)

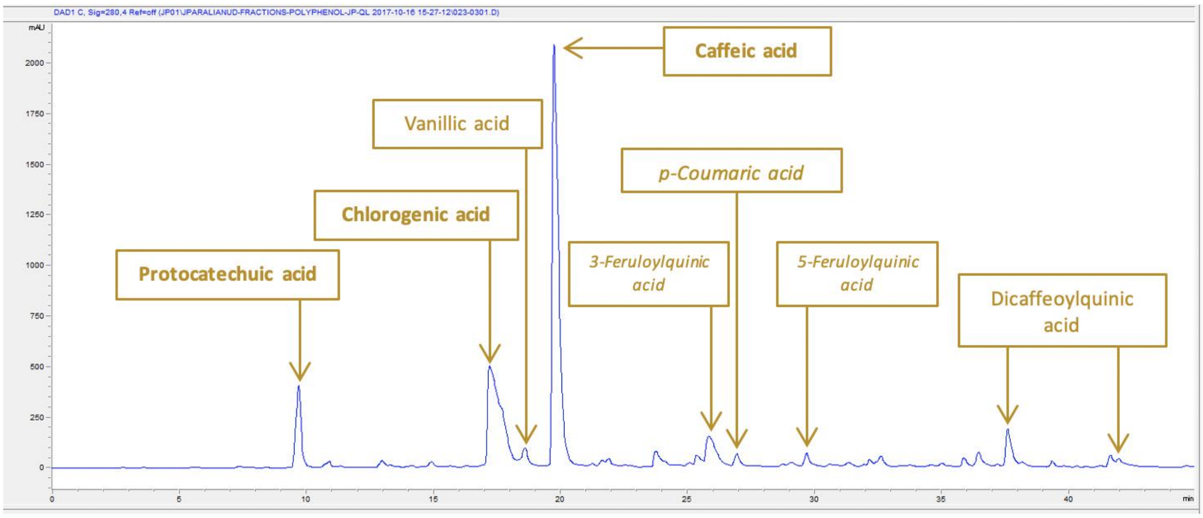

Figure S1. HPLC profiles of FEPC with composition identification.

Table S1. HPLC quantification of FEPC compounds.

	**FEPC (µg/mg ; *w*/*w*%)**
**Protocatechuic acid**	48 ± 1 (**4.8%**)
**Chlorogenic acid**	156 ± 1 (**15.6%**)
**Vanillic acid**	10 ± 1 (**1.0%**)
**Caffeic acid**	153 ± 2 (**15.3%**)
**3-Feruloylquinic acid**	28 ± 2 (**2.8%**)
***p*-Coumaric acid**	4.7 ± 1 (**0.47%**)
**5-Feruloylquinic acid**	4.8 ± 1 (**0.48%**)
**Dicaffeoylquinic acid**	21 ± 2 (**2.1%**)
